# Contextualizing the Neural Vulnerabilities Model of Obesity

**DOI:** 10.3390/nu15132988

**Published:** 2023-06-30

**Authors:** Timothy D. Nelson, Eric Stice

**Affiliations:** 1 Department of Psychology, University of Nebraska-Lincoln, Lincoln, NE 68588, USA; 2 Department of Psychiatry and Behavioral Sciences, Stanford University School of Medicine, Stanford, CA 94305, USA; estice@stanford.edu

**Keywords:** neural vulnerabilities, obesity, context, eating behavior, reward sensitivity, regulation, executive control, inhibitory control, adolescence

## Abstract

In recent years, investigators have focused on neural vulnerability factors that increase the risk of unhealthy weight gain, which has provided a useful organizing structure for obesity neuroscience research. However, this framework, and much of the research it has informed, has given limited attention to contextual factors that may interact with key vulnerabilities to impact eating behaviors and weight gain. To fill this gap, we propose a *Contextualized Neural Vulnerabilities Model of Obesity*, extending the existing theory to more intentionally incorporate contextual factors that are hypothesized to interact with neural vulnerabilities in shaping eating behaviors and weight trajectories. We begin by providing an overview of the *Neural Vulnerabilities Model of Obesity*, and briefly review supporting evidence. Next, we suggest opportunities to add contextual considerations to the model, including incorporating environmental and developmental context, emphasizing how contextual factors may interact with neural vulnerabilities to impact eating and weight. We then synthesize earlier models and new extensions to describe a *Contextualized Neural Vulnerabilities Model of Obesity* with three interacting components—food reward sensitivity, top-down regulation, and environmental factors—all within a developmental framework that highlights adolescence as a key period. Finally, we propose critical research questions arising from the framework, as well as opportunities to inform novel interventions.

## 1. Contextualizing the Neural Vulnerabilities Model of Obesity

In recent years, a framework for conceptualizing obesity risk in the context of specific neural vulnerabilities has emerged [1,2] and provided a useful organizing structure for work in the field of obesity neuroscience. Within this framework, specific individual-level factors, particularly high *reward sensitivity* to high-calorie foods, and low top-down *regulation* abilities (most notably, inhibitory control) are considered critical risk factors for the development of unhealthy eating and, ultimately, obesity. A growing literature documents considerable individual differences in both sensitivity to food rewards and regulation abilities (particularly aspects of executive control, which is also often referred to as executive function), and links these critical neural vulnerability factors to obesogenic eating behaviors and excess weight gain [1,3]. However, the *Neural Vulnerabilities of Obesity* framework, and much of the research informed by this perspective, has given relatively limited attention to contextual factors that may interact with key vulnerabilities to impact eating behaviors and weight gain in important ways. To fill this gap, we propose a *Contextualized Neural Vulnerabilities Model of Obesity*, extending existing theory to more intentionally incorporate the consideration of various contextual factors that are hypothesized to interact with individual reward sensitivity and regulation abilities in shaping eating behaviors and, ultimately, long-term weight trajectories. Our hope is that this extension of the neural vulnerabilities model will provide a useful conceptual framework for increasingly rich and contextualized investigations into the interplay between brain, environment, behavior, and health.

In this paper, we begin by providing an overview of the *Neural Vulnerabilities Model of Obesity* and a brief review of the empirical evidence supporting the model. Next, we suggest opportunities to add contextual considerations to the model, including the incorporation of environmental and developmental context, with an emphasis on how contextual factors may interact with neural vulnerabilities to impact eating and weight trajectories. We then attempt to synthesize earlier models and new extensions to describe a *Contextualized Neural Vulnerabilities Model of Obesity* with three major interacting components – approach factors for unhealthy eating (including food reward sensitivity), top-down regulation abilities (including executive control of attention to food cues, executive control of appetitive response, and emotion regulation), and environmental factors (including availability and cues for unhealthy consumption in the home, neighborhood, family, and peer contexts) – all within a developmental framework highlighting adolescence as a potentially key period. Finally, we propose critical falsifiable research questions arising from the conceptual framework, as well as opportunities to inform novel interventions.

## 2. The Neural Vulnerabilities Model, Extensions, and Supporting Literature

The *Neural Vulnerabilities Model of Obesity* provides a framework for conceptualizing neural factors that may predispose an individual to the over-consumption of calorically-dense foods and excess weight gain. As reviewed by Stice and Burger [1], the extant literature suggests two specific vulnerabilities with particularly consistent links to obesity: *high reward sensitivity*, as reflected in neural hyper-sensitivity to high-calorie foods; and *regulation deficits*, particularly low inhibitory control. These dual vulnerabilities may also work in tandem, as in an *accelerator/brake* metaphor, with high reward sensitivity creating a strong approach motivation toward unhealthy food (the “accelerator”) and regulation serving as a “brake” on excessive consumption. From this perspective, individuals with an overactive accelerator (i.e., too much reward sensitivity), and weak or ineffective brakes (i.e., too little regulation) may be at risk for habitual over-consumption of unhealthy foods. Notably, the factors of reward and regulation—and the idea that they may work in tandem to affect behavior—generally map onto broader neuroscience models of self-regulation failure, such as the *Balance Model of Self-Regulation*, which has also been applied to eating behavior [4,5]. Interestingly, the opposite pattern of low reward responsiveness and very high regulation—not enough accelerator, and too much brake—may characterize individuals at risk of anorexia nervosa (AN). Specifically, women with, or recovered from, AN show a weaker responsivity of the reward and motivational regions (e.g., insula, striatum, anterior cingulate cortex [ACC]) to images and tastes of high-calorie foods versus the healthy controls [6,7,8,9]. In addition, women with, or recovered from, AN show greater recruitment of the inhibitory regions (e.g., dorsolateral prefrontal cortex [dlPFC], inferior frontal cortex) in response to high-calorie food images and obesity-related words than do healthy controls [10,11], and they also show less delay discounting for money than do controls, implying elevated self-control [12,13].

Further extending the neural vulnerabilities model, researchers have developed increasingly sophisticated frameworks focusing on how specific regulatory deficits can lead to unhealthy eating patterns and excess weight gain. Hall and Marteau [14] proposed a conceptual model highlighting *executive control* deficits as a key mechanism impacting a wide variety of health behaviors, obesity, and overall health risks in adults. In a developmental extension of this model, Nelson and colleagues [15] detailed how deficits in specific components of executive control (including working memory, inhibitory control, and flexible shifting) in adolescence could contribute to breakdowns of attentional, behavioral, and emotional control, leading to unhealthy eating and weight trajectories. Taken together, these frameworks proposing key roles for a variety of regulatory abilities, particularly those falling under the umbrella of “executive control”, extend the neural vulnerabilities model, which has focused primarily on inhibitory control deficits, to include related abilities with a potential relevance to obesity.

A substantial and growing empirical literature supports the role of reward sensitivity and regulation as key neural vulnerability factors for obesity. First, numerous studies have linked a high reward sensitivity with a greater risk for unhealthy food consumption and obesity (see [16] for review). Particularly pertinent to neural vulnerabilities, elevated reward region response to both tastes of high-calorie food and cues for high-calorie food have been associated with overeating and/or obesity in prospective studies. Specifically, elevated responsivity of brain regions implicated in reward valuation (striatum, orbitofrontal cortex) to high-calorie food images and cues has predicted future unhealthy weight gain in longitudinal studies with adolescents and young adults [17,18,19,20]. Along these lines, we recently found that elevated activation of reward regions (e.g., caudate and putamen) upon receiving sips of a chocolate milkshake significantly predicted greater weight gain over a one-year period in a large sample drawn from several longitudinal studies of adolescents and adults [21]. Second, a large body of literature has found associations between specific deficits in top-down regulation abilities and dietary and obesity risk. For example, although results have been mixed, studies spanning the developmental spectrum have reported significant correlations between executive control (and related abilities) and unhealthy eating behaviors and/or obesity (see [22,23], for reviews). Moreover, while the majority of this literature has focused narrowly on deficits in inhibitory control (see [1], for review), a growing number of studies have linked other aspects of executive control (such as working memory or flexible shifting; refs. [3,24,25]), or more comprehensive measures incorporating multiple executive control abilities [26,27], to eating and weight outcomes. Third, a more limited set of studies has examined *interactions between reward sensitivity and regulation* in predicting diet and weight trajectories. For example, in a rare study explicitly testing the interaction between the relative reinforcing value of food and inhibitory control, Loch and colleagues [28] reported that this interaction did not significantly predict the change in adiposity over three years in a sample of children and adolescents. However, rigorous large-sample studies, leveraging neural measures to explore interactions between reward sensitivity and regulation in predicting eating behavior and obesity trajectories, are needed.

## 3. Adding Context to the Neural Vulnerabilities Model

Although the Neural Vulnerabilities of Obesity and related models have guided valuable studies explicating the role of reward sensitivity and top-down regulation in obesity risk, this framework, and much of the research informed by it, gives limited attention to contextual factors. Such factors, including environmental and developmental context, are potentially important in achieving an even more sophisticated understanding of individual risk of unhealthy eating and obesity, which could guide novel interventions. Specifically, as we discuss below, contextual factors may confer risk not only through their direct effects on weight status (which are well-documented in the literature; e.g., refs. [29,30]), but also in how such factors potentially *interact* with neural vulnerabilities of reward sensitivity and top-down regulation to impact weight trajectories. We highlight the particular promise of integrating environmental and developmental contextual factors into the neural vulnerabilities framework.

*Environmental Context.* Environmental factors could be critical in creating the context in which reward and regulation operate. For example, in highly obesogenic *home and neighborhood environments*—characterized by ubiquitous access to, and cues for, unhealthy consumption—the impact of specific neural vulnerabilities (i.e., high reward, low top-down regulation) could be amplified. Individuals who are highly sensitive to food reward may be especially vulnerable to the consumption cues in such environments, including being drawn to attend to and consume highly appetizing (but nutritionally-poor) foods. Relatedly, individuals with poorer top-down regulation abilities may struggle to direct their attention and behavior in healthy ways when confronted with an obesogenic environment. For example, low executive control of attention may lead to over-attending to food stimuli, making it especially difficult to disengage from tempting stimuli when they are prevalent in the environment [15,31]. Further, deficits in response inhibition may be more likely to lead to impulsive and unhealthy consumption when access to unhealthy foods is easy and immediate. Conversely, in less obesogenic environments, temptation and the need for top-down regulation may be substantially lower, thus moderating the impact of these vulnerabilities. While a large and growing literature has documented the main effects of home, neighborhood, and school environments on diet and weight status, very little research exists exploring *interactions* between individual neural vulnerabilities and the environmental context. Integrating environmental factors into the neural vulnerabilities framework could help guide such research by contextualizing the roles of reward and regulation within the physical environment.

Food insecurity represents a unique environmental factor that may interact with neural vulnerabilities to impact eating and obesity trajectories. The uncertainty that comes with not having reliable access to food could distort reward and regulatory processes, with concerns focusing mostly on consumption whenever food is available, with less attention given to the nutritional quality of the food (see [32] for a review of associations between food insecurity and dietary quality). Further, regulation of portion size may become a less salient goal in the context of food insecurity; rather, such circumstances may encourage overeating whenever food is available, thus enhancing the rewarding value of food while undermining regulation abilities. Such processes may contribute to the paradoxical link between food insecurity and obesity [33] and must be understood as an interaction between context and vulnerability factors.

In addition to the physical environment, the *social or relational environment* in which an individual is embedded creates a critical context for eating and obesity trajectories. Specifically, the behaviors, attitudes, and goals of individuals in close proximity to, and who regularly interact with, the focal person could have a considerable impact in shaping the context in which eating occurs. Families play a critical role in establishing norms around eating. For example, parents who model eating dessert after every meal, and encourage children to do the same, influence the context in which food reward and regulation develop and are deployed. Additionally, friends, partners, and other peers such as roommates or co-workers could be important in supporting or not supporting health goals. For example, having a partner who is supportive of pursuing healthy eating and exercise goals could help mitigate high food reward sensitivity and reduce regulatory demands by helping maintain a home environment that is conducive to these goals. In contrast, living with someone who does not support health goals – for example, by consistently bringing home energy-dense/nutrition-poor foods – may exacerbate neural vulnerabilities by increasing temptation in the home environment. And while the main effects of such social factors on diet and obesity are relatively well-documented (see [34], for review), the ways in which these factors *interact* with individual reward sensitivity and regulation abilities to predict unhealthy weight gain has been largely overlooked and represents an opportunity to integrate the critical context into research guided by the neural vulnerabilities framework.

Just as physical and social environments create the “external context” in which neural vulnerabilities exist, there is also an “internal context” of individual-level characteristics that could interact with reward sensitivity and regulation. Perhaps most notably, *negative affect* could create an internal context in which reward and regulation operate. Depression, anxiety, and loneliness, for example, are prevalent manifestations of negative affect that likely have complex interplays with reward processing, regulation, and eating [4,35]. While the literature surrounding the main effects of negative affect on eating behaviors is considerable (see [36], for review), the interaction between negative affect and neural vulnerabilities in the context of diet and obesity is less frequently studied. The internal context of high negative affect may create conditions that ultimately lead to enhancing the rewarding value of food, both immediately and over time. For example, Wagner and colleagues [35] found that inducing negative affect increased reward region activation in response to appetizing food images compared to a neutral mood control condition. Further, negative affect may create a drive to eat appetizing (yet ultimately unhealthy) foods as a way of combatting negative emotions (a “food as self-medication” hypothesis). In their meta-analysis, Cardi and colleagues [37] reported that experimentally-induced negative mood was associated with greater food consumption. There is also evidence that experimentally-induced stress increases reward region response to milkshake tastes [38]. Because such energy-dense foods are often immediately gratifying, their consumption *in the context of negative affect* could be negatively reinforced through the temporary reduction of unpleasant emotions, thus increasing this behavior when negative emotions are experienced. Along these lines, Ranzenhofer and colleagues [39] assessed negative affect before and after a laboratory meal in a sample of adolescent girls with loss of control eating, and they found that a higher pre-meal negative affect was associated with greater snack and dessert intake, and that there was a significant decrease in negative affect from pre-meal to post-meal.

Relatedly, when negative affect is repeatedly paired with high calorie consumption, the experience of negative affect can theoretically become a cue for eating, even in the absence of hunger, which increases obesity risk. Further, individuals with poor top-down regulation abilities may be especially at risk of falling into these maladaptive processes because they struggle to regulate and respond to negative emotions in healthier ways [15]. Findings from our longitudinal research on developing executive control and obesity risk hint at such a process. We have found that the interaction between negative affect temperament and executive control predicts both unhealthy eating (including high sugar and sugar-sweetened beverage intake [27]) and BMI gain across adolescence [40], with deficits in executive control being particularly predictive in the context of high negative affect. Further, Yang et al. [41] reported an interaction between negative affect and reward region response to appetizing foods, with high negative affect amplifying the association between neural response to food and future weight gain.

*Developmental Context.* In addition to environmental context, adding *developmental context* to the neural vulnerabilities framework could be valuable. Reward sensitivity, regulation abilities, and eating behaviors all have unique developmental trajectories [42,43,44], and it is essential to consider the dynamic interplay between these factors at key points in development. Although studies have found associations between neural vulnerabilities and weight from early childhood through adulthood [17,26], we argue that adolescence may be a particularly critical period for consideration within the neural vulnerabilities framework given its importance in the development of eating habits and weight trajectories, as well as it being a time in which the interplay between reward, regulation and the environment may be especially relevant. Adolescence is a unique developmental period characterized by increasing autonomy, which “raises the stakes” for health behaviors and “presses” the adolescent to deploy top-down regulation to direct behavior toward health. Adolescence is also a time of heightened reward sensitivity and emotional reactivity but still-developing regulatory abilities [45], creating a potential developmental imbalance that undermines the top-down control of attention and behavior [46]. Further, adolescents have limited control over their food environments (e.g., they do not typically grocery-shop or decide where they live or go to school), but these environments could be critical in shaping their eating behavior. Because of the combination of increasing health behavior autonomy, heightened reactivity, immature regulation abilities, and vulnerability to environmental context, adolescence represents a unique developmental context in which to understand obesity risk from a contextualized neural vulnerabilities perspective. The importance of this developmental period is further highlighted by the elevated rates of obesity relative to earlier childhood and escalating rates over time [47], as well as generally low dietary quality for adolescents relative to other age groups [44]. Relatedly, adolescence may be an ideal time for intervention to prevent obesity before processes that lead to the habitual overvaluation and overconsumption of energy-dense foods become entrenched. However, much of the extant literature on reward, regulation and obesity has focused on adults, and thus misses critical developmental context.

## 4. A Contextualized Neural Vulnerabilities Model of Obesity

**Figure 1:**
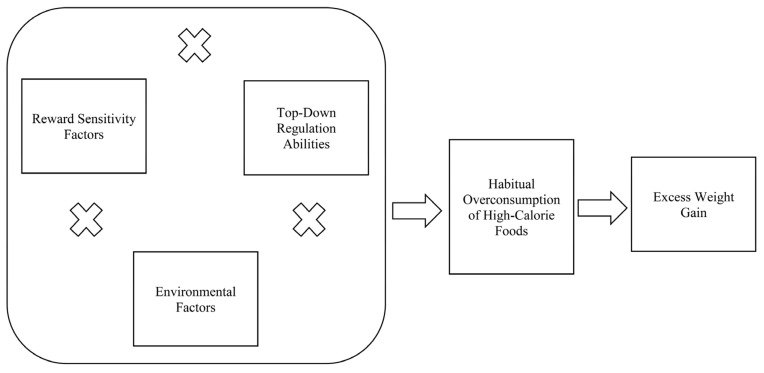
Contextualized Neural Vulnerabilities of Obesity Model.

Integrating the considerations discussed above, we propose a *Contextualized Neural Vulnerabilities Model of Obesity* (see Figure 1). The model is comprised of three major components—reward sensitivity factors, top-down regulation abilities, and environmental factors—all of which are embedded within a developmental context. While each component is expected to have direct effects on eating behaviors and weight trajectories, the novel contribution of this model is in its focus on potential *interactions between components* to create a more contextualized conceptualization of obesity risk that will inform new directions in research, as well as targeted prevention and intervention.

The three components of the *Contextualized Neural Vulnerabilities Model*, as well as their interactions, are depicted in Figure 1. First, reward sensitivity factors represent individual differences in the reinforcing value of energy-dense/nutrition-poor food, including those high in fat or added sugar, and highly processed foods. It is expected that individuals who are highly sensitive to the rewarding qualities of such foods will be motivated to consume unhealthy foods at a high rate, thus contributing to habitual over-consumption and risk for excess weight gain. An emerging obesity neuroscience literature suggests that elevated reward region response in a variety of situations—including viewing images of high-calorie foods, anticipation of receiving high-calorie food tastes, and actual high-calorie food tastes—may all be relevant in predicting future weight gain [1,21]. Furthermore, it may be useful to distinguish between early-emerging, biologically-determined individual differences in neural responsivity to high-calorie foods and reward region responses to cues that have come to be associated with hedonic pleasure over time. While the former may represent a static individual vulnerability, the latter is conceptualized as more of an *emergent risk factor* that develops through a conditioning *process*. This process may unfold over time with repeated pairing of cues with hedonic pleasure, resulting in an increased attention and reactivity to cues in reward regions, which in turn increases the risk of overeating and weight gain.

Second, regulation factors refer to an individual’s ability to exert top-down regulation to direct attention and behavior in intentional ways. Considerable individual differences in such abilities (often conceptualized as executive control or some of its components) have been documented across the developmental spectrum [48,49,50,51], and deficits in specific abilities (e.g., inhibitory control, working memory, cognitive flexibility) could impact an individual’s ability to achieve health goals (see [14,15], for discussion). By far the most robust literature in this area has focused on low inhibitory control as a risk factor for future weight gain. Inhibitory control deficits in response to high-calorie foods in delay-discounting tasks, which reflect a bias toward immediate rewards, have reliably predicted future weight gain [52,53,54,55]. Similar results have emerged from studies using self-report measures of inhibitory control [56,57,58]. Individuals with inhibitory control deficits also show a poorer response to weight loss treatment and poorer weight loss maintenance [59,60,61]. Further, individuals who showed less recruitment of inhibitory control regions (dorsolateral prefrontal cortex) during a delay discounting task showed significantly less weight loss in response to weight loss treatment [60] and less weight loss maintenance over a one-year follow-up [61]. Moreover, while the majority of the literature on regulation and obesity has focused on inhibitory control, specifically, other aspects of executive control, particularly working memory and flexible shifting, have begun to receive more attention, both conceptually [14,15] and empirically (see [3,24], for relevant reviews). Therefore, recognizing the potential role of a range of regulatory abilities in eating and obesity, our model includes a broad conceptualization of top-down regulation factors as potentially important neural vulnerabilities for obesity.

One potentially important distinction here is between general top-down regulation (which occurs across a wide variety of stimuli and contexts) and top-down regulation that occurs within the specific context of food. Research rigorously examining the relative impact of these distinct types of regulation is currently lacking, but it is possible that both forms could be relevant for obesity, with general regulation abilities creating a broad foundation for a more food-specific regulation that develops over time. For example, healthy-weight adolescents with versus without a parental history of obesity show greater reward region response to both monetary reward and tastes of high-calorie foods [62,63]. Theoretically, a general deficit in inhibitory control increases the likelihood of beginning to consume high-calorie foods, and the habitual intake of high-calorie foods may create even greater deficits in inhibitory control because it contributes to greater reward region responsivity to cues for high-calorie foods, which is a risk factor for future weight gain [64]. That is, the process of overeating appears to increase a key neural vulnerability factor for future unhealthy weight gain. Research has captured this incentive sensitization conditioning process wherein a previously neutral visual cue acquires motivational significance when it is repeatedly paired with tastes of chocolate milkshake, resulting in an increase in caudate, putamen, and ventral pallidum response to the visual cue [65]. Consistent with expectations, individuals who showed the greatest increase in striatal responsiveness to the cue showed a significantly higher future weight gain [65].

Third, environmental factors include a wide range of contextual considerations that shape diet and weight trajectories. In our conceptualization, these include both “external context” factors and “internal context” factors. External context refers to environments outside the individual such as key physical food environments (e.g., home, neighborhood, school, work) and social or relational systems (e.g., parents, peers, partners), which together comprise the food environment that surrounds the individual on a daily basis [66]. Important aspects of the external food environment include the availability and accessibility of healthy and unhealthy foods (e.g., what foods are easily accessible within the home and community [67]) and environmental cues for consumption (e.g., advertisements for fast food, consumption patterns of others around the individual, social norms around eating [68]). Internal context refers to contextual influences within the individual that can affect dietary behavior, most notably the presence of negative affect (e.g., depression, anxiety, loneliness), which can become a cue for eating high-calorie foods [35]. Broadly speaking, obesogenic environmental contexts—characterized by the high availability of unhealthy foods and various internal and external cues for their consumption—may “set the stage” for habitual unhealthy eating and long-term excess weight gain.

In addition to the main effects of reward sensitivity, regulation abilities, and environment factors, several interactions between these model components are hypothesized to impact dietary and weight outcomes. Existing models have already highlighted some potential roles of reward x regulation interactions, with a greater reward sensitivity expected to create a stronger drive toward overeating, thus requiring stronger regulation abilities to meet dietary goals [4]; however, research rigorously testing such interactions is limited. Our contextualized extension also proposes critical interactions between key neural vulnerabilities (reward sensitivity and regulation abilities) and environmental factors. Individuals with a high food reward sensitivity may be particularly vulnerable to food environments in which highly appetizing foods are readily available and consumption cues are ubiquitous, creating temptation for consumption that may be inconsistent with dietary goals (a reward x environment interaction). Similarly, successfully navigating such challenging environments may require stronger regulation abilities, with obesogenic environments exposing deficits in regulation more than healthy environments which may buffer the risk in individuals with weaker regulation abilities (a regulation x environment interaction). Taken together, these complex interactions suggest that dietary and obesity risk emerge from the *combination* of risk factors in context, with high reward sensitivity creating a motivational approach toward unhealthy foods, the environment creating opportunities for consumption of such foods, and then low regulation resulting in breakdowns in intentional control over eating in the presence of both motivation and opportunity.

Finally, the entire proposed model is further contextualized within a developmental perspective that recognizes that the three model components unfold in unique ways across different developmental periods. Certain regulation abilities, such as executive control, follow an extremely protracted developmental course, with critical growth periods in preschool and adolescence, and maturation continuing into the 20s, before declining later in adulthood [43,69]. Reactivity to rewards may also change across development [42], although normative patterns and individual differences in the trajectories of food reward sensitivity specifically are not well-understood. Similarly, environmental factors must be considered within the developmental context as the centrality of certain relationships (e.g., parents versus peers) and environments (e.g., home versus school, community, or workplace) changes over time. Furthermore, all of these considerations occur against the backdrop of significant shifts in autonomy over health behaviors from childhood to adolescence to adulthood, which may influence the impact of different risk factors. While our contextualized model may be usefully applied to diverse developmental periods, we highlight adolescence as a particularly important time to consider the interactions between reward sensitivity, regulation, and environment. As noted, adolescence is a time when surging reward sensitivity may overwhelm still-developing regulatory abilities, particularly in the context of emerging health behavior autonomy and environmental cues for unhealthy consumption. This combination may make adolescence an especially vulnerable period, particularly for those with deficits in regulation.

## 5. Pathways to Obesity

One implication of the proposed *Contextualized Neural Vulnerabilities Model* is that there may be diverse pathways to obesity for individuals with different combinations of neural and contextual risk factors. For example, high food reward sensitivity in an environment with easy access to high-calorie foods may overwhelm even relatively strong regulatory abilities, leading to over-consumption. Alternatively, deficits in regulation abilities may combine with cues in the social environment that encourage overeating to increase risk for excess weight gain. However, while we can envision numerous starting points on the path to obesity, we propose that diverse pathways converge on the common process of the habitual over-consumption of high-calorie foods (see Figure 1). Habitual over-consumption, in turn, triggers a conditioning process resulting in the overvaluation of anticipated rewards from high-calorie foods, thus increasing attention and reactivity to these foods and related cues. Once this process of over-consumption and overvaluation begins, it can be difficult to interrupt and reverse, leading to long-term risk of excess weight gain. From this perspective, any factors that lead to the initiation of overeating can kick off a cascade of processes that heighten food rewards, undermine regulation, and entrench overeating patterns. If such a model were supported, the implications for intervention, particularly addressing “upstream” risk factors to prevent initial over-consumption, would be clear.

## 6. Informing a Contextualized Neural Vulnerabilities Model of Obesity Agenda

The proposed model informs a novel research agenda in contextualized obesity neuroscience, raising key research questions to test and refine components of the model. In addition to exploring the main effects of the three components on eating behavior/weight gain (with innovative research programs in these areas already underway), there is a need for studies incorporating all three components together. Such research could have two purposes. First, because much of the research exploring elements of the neural vulnerabilities model has examined individual components in isolation, studies incorporating multiple elements could help determine which components are the most impactful and, therefore, the most promising targets for intervention. Second, our model points toward explicating the *interactions* between different components and their impact on eating behavior and weight gain. Below, we propose some research questions that could be particularly informative in testing and refining the model, and ultimately informing contextualized approaches to intervention and prevention. For each, we offer specific predictions based on our model. Findings consistent with our predictions would support the model, whereas findings that contradict our hypotheses would suggest the need to revise the model.

*Reward x Regulation*. Research question: How is the effect of top-down regulation abilities on diet/weight trajectories moderated by individual differences in reward sensitivity? Prediction: We expect a significant reward x food regulation interaction, such that regulation deficits are most predictive of unhealthy dietary and weight trajectories *in the context of high reward sensitivity*. This prediction is based on the idea that strong regulation is needed in the context of high reward sensitivity (which creates a strong approach to unhealthy foods), and that the combination of high reward sensitivity and low regulation is especially conducive to developing unhealthy eating.

*Environment x Regulation.* Research question: How is the effect of top-down regulation abilities on diet/weight trajectories moderated by individual environmental factors (both “external” and “internal”)? Prediction: We expect a significant environment x food regulation interaction, such that regulation deficits are most predictive of unhealthy dietary and weight trajectories *in the context of high obesogenic external environmental factors* (e.g., high availability of unhealthy foods in the home and neighborhood; high parental modeling of the consumption of energy-dense foods; low partner or friend support for health behavior goals). Similarly, we expect a significant environmental x food regulation interaction, such that regulation deficits are most predictive of unhealthy dietary and weight trajectories *in the context of internal factors such as high negative affect* (e.g., high temperamental negative affect; high symptoms of depression, anxiety, and/or loneliness). These predictions are based on the idea that strong regulation is needed in the context of highly obesogenic environments (which creates the opportunity and cues for unhealthy consumption), and that the combination of a highly obesogenic environment and low regulation is especially conducive to developing unhealthy eating.

***Reward x Environment.*** Research Question: Does the combination of high reward sensitivity and an obesogenic environment (external and internal) result in an additive or multiplicative risk of unhealthy dietary and weight trajectories? Prediction: Environmental factors (both external and internal) will moderate the effect of individual differences in reward sensitivity on the prediction of dietary and weight trajectories, such that more obesogenic environments will exacerbate the effect of a high reward sensitivity on eating and weight outcomes. This prediction is based on the idea that high reward sensitivity will create a strong motivation to consume unhealthy foods and that obesogenic environments provide the opportunity for such consumption. We also expect an interplay between food environments and reward sensitivity, such that living in a more obesogenic environment, particularly during critical developmental periods such as adolescence, may condition individuals to overvalue energy-dense foods. This emergent sensitivity to food cues is expected to be a risk factor for future overeating and weight gain.

***Food-Specific versus General Regulation.*** In addition to the proposed interaction analyses, it is also critical to empirically address the relative contributions of food-specific regulation versus general (i.e., non-food) regulation abilities in predicting dietary and weight trajectories. Studies including rigorous measures of both food-specific and general regulation in predicting diet and weight trajectories are currently lacking, leaving a potentially important question regarding optimal intervention targets unanswered. On the one hand, deficits in food-specific inhibitory control are highly correlated with generic inhibitory control *(r* = 0.70) [28], suggesting that the two constructs are highly related, as one would expect. However, there is also evidence that response inhibition training with high-calorie foods produces weight loss compared to a control condition that completed the same response inhibition training with non-food images [70,71,72]. Future research should further explicate the roles of food- versus non-food regulation to inform targeted treatment approaches.

***Developmental Studies.*** Across the diverse research questions raised, there is a critical need for studies that are informed by a developmental perspective. For example, it would be highly informative to conduct research that charts the developmental trajectories of the contextualized neural vulnerabilities model components over time. We know little about how constructs such as food reward sensitivity and food-specific regulation change across development, and if there are critical periods during which these factors are particularly influential on diet and weight trajectories. These gaps limit our ability to identify key periods when targeted intervention may be most impactful in modifying risk factors and, in turn, changing health trajectories. As noted, we have proposed conceptual reasons for why adolescence might be a particularly important period for observational longitudinal studies (and possibly intervention trials), but rigorous studies of the contextualized model components in this development stage are sparse.

***Exploring Differential Risk Pathways to Obesity.*** It is also important to consider the possibility that individuals may take qualitatively different risk pathways to obesity, rather than all experiencing a single risk pathway. Answering this question may require that we move away from ordinary least squares analyses that seek to fit a single model to the data, and use machine-learning techniques that can detect qualitatively different risk pathways to negative health and mental health outcomes. We have argued that classification tree analyses are particularly well suited to uncovering qualitatively different risk pathways with prospective data [73]. Although a literature search did not identify any prospective study that has used classification tree analyses to predict the future onset of obesity, one study did use it to predict the future onset of binge eating over a two-year follow-up among adolescent girls who initially did not report binge eating [74]. Classification tree analyses select the most potent risk factor and identify the cut-point that shows the greatest potency for differentiating who will, versus will not, experience onset of the outcome (binge eating). Overvaluation of weight/shape for determining self-worth was the first predictor: 20% of girls in the upper 50% of weight/shape overvaluation showed onset of binge eating, whereas only 2% of those in the lower 50% of the distribution showed binge eating onset. The fact that weight/shape overvaluation emerged first signals that it had greater predictive power than all of the other variables included in the model, which included dieting, BMI, body dissatisfaction, pressure for thinness, depressive symptoms, self-esteem, emotional eating, modeling of eating-disordered behaviors, and low social support. Among girls with low weight/shape overvaluation, 9% of those in the upper 25% of depression scores showed binge eating onset, versus 0% for those with lower depression. Among girls with high weight/shape overvaluation, 27% of those with a BMI of 18 or greater showed binge-eating onset, versus 0% for those with a lower BMI. Among girls with a high weight/shape overvaluation and a BMI > 18, 42% showed onset of binge eating if they were in the upper 40% of dieting versus 16% for those with lower dieting. Thus, the results revealed a four-way interaction between weight/shape overvaluation, depression, BMI, and dieting. Findings suggested that an elevated BMI amplified the predictive relation between weight/shape overvaluation and binge eating onset. This amplifying interaction indicates that the attitudinal risk factor of appearance overvaluation only operates among adolescent girls who have an age- and gender-adjusted BMI that places them in the slightly overweight range; thus, for girls who conform to the thin ideal, weight/shape overvaluation was not associated with binge eating onset. The results indicated another amplifying interaction, wherein dieting increased the predictive effects of the combination of weight/shape overvaluation and elevated BMI, with almost half the participants with this triple confluence of risk factors showing binge eating onset. Last, the results suggested an alternative pathway interaction, wherein among adolescent girls with lower weight/shape overvaluation, elevated depression emerged as a risk pathway, theoretically because depression increases the reward value of food or people turn to eating for mood improvement. That is, this classification tree suggested two qualitatively distinct risk pathways to binge eating onset; one involving a combination of weight/shape overvaluation, elevated weight, and dieting, and another involving elevated depression.

It would be useful for future prospective studies to apply classification tree analyses to the prediction of obesity onset over a multi-year follow-up because this might allow us to identify qualitatively different risk pathways to obesity. Results may inform more precision medicine-based prevention programs and treatments.

***Randomized Controlled Trials.*** Finally, there are notable opportunities for research that develops and tests interventions that address key components of the model, particularly during critical developmental periods such as adolescence. Randomized controlled trials, including randomized prevention trials, may be an ideal follow-up for confirming findings from prospective observational risk factor studies (such as those suggested above), and determining the impact of targeted interventions based on a contextualized neural vulnerabilities model framework. Below, we provide a more detailed discussion of potential prevention and intervention implications.

## 7. Limitations and Opportunities for Further Development

Like all models, the contextualized neural vulnerabilities model described in this paper has limitations that should be acknowledged and considered as opportunities for further model refinement. First, our model does not explicitly focus on the biological foundations of neural vulnerabilities or the biological processes associated with obesity that could exacerbate neural vulnerabilities. For example, inflammatory processes, reduced cerebrovascular function, and disruptions in the gut microbiome could each further compromise cognitive and regulatory processes in ways that create additional risk for unhealthy eating and weight gain over time. Such complex bidirectional effects between neural vulnerabilities and biological processes associated with obesity may be particularly important over the long-term once unhealthy weight gain begins. Research that explicitly explores such processes and integrates the findings into the model would be useful. Second, our model focuses on two specific neural vulnerabilities, high reward sensitivity and regulatory deficits, because these factors have the most research support linking them to obesity risk; however, other neural vulnerabilities may also be relevant. As additional factors are identified in the literature as meaningful neural vulnerabilities for obesity, these factors should be integrated into the contextualized model with a focus on how they interact with key contexts to impact eating and weight trajectories.

## 8. Implications for Prevention and Intervention

The proposed model, if supported, could inform novel obesity prevention and intervention strategies. Specifically, if the three components are impactful, either via direct effects or through their interactions, this could create multiple points for intervention. We briefly present here some ideas for possible prevention and intervention targets.

If research supports a key role for food reward sensitivity in dietary and obesity risk, interventions could focus on reducing such sensitivity via targeted training. One potential way of reducing elevated reward region response to tastes of high-calorie foods could be to use dietary supplements that block certain taste receptors (e.g., sweet taste receptors), although rigorous trials examining the efficacy of such supplements in preventing weight gain are needed. This review also suggests that reducing the reward region response to cues for high-calorie foods might be useful in preventing future weight gain. Randomized trials have found that computer-training in which participants are cued to make a behavioral response to pictures of fruits and vegetables and to inhibit a behavioral response to high-calorie foods that the participant reports overeating over a 4- to 6-week period significantly reduces reward region response to pictures of high-calorie foods, palatability ratings of and willingness to pay for high-calorie foods, and produces objectively-measured body fat loss that persists through 2-year follow-up [70,71,72]. Reducing food reward sensitivity may be particularly beneficial for individuals with deficits in regulation because reducing the motivational drive to consume energy-dense foods could lower the demand on regulation abilities.

Enhancing regulation abilities is another possible strategy for prevention and intervention. If food-specific regulation proves particularly important, training could seek to build these specific abilities (rather than more general, non-food regulation) in the context of realistic food stimuli. Training interventions to down-regulate appetitive responses to energy-dense foods could leverage technologies such as virtual reality to create realistic, contextualized opportunities to practice food regulation. Further, interventions focusing on supporting skill *application in context* could provide prompts to remember health goals and direct attention away from tempting but unhealthy cues in the environment to increase the chances of enacting healthy decisions in difficult situations. Relatedly, interventions that focus on developing health goals and enhancing motivation to implement those goals (e.g., by creating cognitive dissonance regarding lifestyle behaviors that contribute to unhealthy weight gain [70,71,72]), even when confronted with tempting food stimuli, could support efforts to deploy regulation abilities toward health. Alternatively, for individuals with significant deficits in specific regulation abilities, compensatory strategies or “work arounds” might be helpful. For example, for those with poor working memory, targeted messages reminding the individual of their health goals, either periodically or in specific tempting situations, could help to keep such goals top of mind and encourage decisions that are consistent with these goals.

Finally, interventions to modify the external and internal food environment contexts could be useful. A number of interventions already seek to change external contexts such and home, school, work, and neighborhood food environments—with a focus on reducing the availability of and cues for unhealthy foods and increasing the accessibility of healthy alternatives—and have produced positive effects on weight outcomes [75,76]. Such strategies could be especially important for individuals with high food reward sensitivity or regulation deficits that put them at risk for unhealthy consumption, as reducing the opportunities to consume energy-dense foods could help mitigate these risks. Modifying internal contexts by reducing negative affect that can drive emotional eating could also help reduce the demands on food regulation. Alternatively, because environments may not always be modifiable, individuals who encounter particularly challenging food environments may benefit from extra support to enhance food regulation or reduce food reward sensitivity to increase their chances of successfully meeting health goals despite environmental challenges.

To summarize, there is potential for creating novel, comprehensive, multi-component intervention approaches that address all three components of the *Contextualized Neural Vulnerabilities Model of Obesity*. Furthermore, informed by longitudinal observational risk studies with an emphasis on explicating processes and pathways, it may be possible to create targeted screening processes that identify individuals at heightened risk for developing habitual unhealthy eating and obesity. At a minimum, screening could involve assessments of the three model components, and individuals with significant risk in one or more areas could be candidates for targeted prevention efforts. Understanding unique risk pathways may also create opportunities for personalizing prevention efforts by identifying combinations of risk factors and tailoring intervention components to the unique needs of the individual. To facilitate such personalized approaches, it would be necessary to develop a “menu” of effective intervention modules for addressing each of the three main components, and then intervention could be personalized by picking and choosing which intervention strategies best fit a particular case.

## Data Availability

Not applicable.
